# Vaccine Increases the Diversity and Activation of Intratumoral T Cells in the Context of Combination Immunotherapy

**DOI:** 10.3390/cancers13050968

**Published:** 2021-02-25

**Authors:** Lucas A. Horn, Kristen Fousek, Duane H. Hamilton, James W. Hodge, John A. Zebala, Dean Y. Maeda, Jeffrey Schlom, Claudia Palena

**Affiliations:** 1 Laboratory of Tumor Immunology and Biology, Center for Cancer Research, National Cancer Institute, National Institutes of Health, Bethesda, MD 20892, USA; lucas.horn@nih.gov (L.A.H.); kristen.fousek@nih.gov (K.F.); duane.hamilton@nih.gov (D.H.H.); hodgej@mail.nih.gov (J.W.H.); schlomj@mail.nih.gov (J.S.); 2 Syntrix Pharmaceuticals, Auburn, WA 98001, USA; jzebala@syntrixbio.com (J.A.Z.); dmaeda@syntrixbio.com (D.Y.M.)

**Keywords:** cancer vaccine, combination immunotherapy, TCR diversity

## Abstract

**Simple Summary:**

Innovative strategies to reduce immune suppression and activate tumor-specific immunity are needed to help patients who do not respond or become resistant to immune checkpoint blockade therapies. In this study, we demonstrate that the addition of a cancer vaccine targeting a tumor-associated antigen to a checkpoint inhibitor-based immunotherapy induces greater numbers of proliferative, activated, and cytotoxic tumor-infiltrating T cells, leading to improved antitumor activity in tumors otherwise resistant to immunotherapy. Our results provide the rationale for the addition of cancer vaccines in combination immunotherapy approaches being evaluated in the clinic.

**Abstract:**

Resistance to immune checkpoint blockade therapy has spurred the development of novel combinations of drugs tailored to specific cancer types, including non-inflamed tumors with low T-cell infiltration. Cancer vaccines can potentially be utilized as part of these combination immunotherapies to enhance antitumor efficacy through the expansion of tumor-reactive T cells. Utilizing murine models of colon and mammary carcinoma, here we investigated the effect of adding a recombinant adenovirus-based vaccine targeting tumor-associated antigens with an IL-15 super agonist adjuvant to a multimodal regimen consisting of a bifunctional anti-PD-L1/TGF-βRII agent along with a CXCR1/2 inhibitor. We demonstrate that the addition of vaccine induced a greater tumor infiltration with T cells highly positive for markers of proliferation and cytotoxicity. In addition to this enhancement of cytotoxic T cells, combination therapy showed a restructured tumor microenvironment with reduced T_regs_ and CD11b^+^Ly6G^+^ myeloid cells. Tumor-infiltrating immune cells exhibited an upregulation of gene signatures characteristic of a Th1 response and presented with a more diverse T-cell receptor (TCR) repertoire. These results provide the rationale for the addition of vaccine-to-immune checkpoint blockade-based therapies being tested in the clinic.

## 1. Introduction

Immune checkpoint blockade therapies have led to successful and durable responses in patients with various tumor types [1,2]. Despite this great success, only a small percentage of patients with solid malignancies experience complete responses with antibodies directed against programmed cell death protein 1 (PD-1), programmed death ligand 1 (PD-L1), or cytotoxic T-lymphocyte associated protein 4 (CTLA-4) as monotherapies [3]. Expanding knowledge of the mechanisms of immunoregulation and resistance to immune checkpoint blockade therapy has allowed researchers to better formulate combinations of drugs aimed at simultaneously targeting the numerous inhibitory factors and cell types responsible for tumor-induced immune suppression and treatment failure [4,5].

Immunologically “cold” or non-inflamed tumors present with a series of unique problems that cannot be overcome by immune checkpoint blockade or modification of the tumor microenvironment (TME) [6,7], including deficiencies in T-cell recognition of tumor antigens, dendritic cell priming, and lymphocyte homing to the tumor tissue. One approach being investigated to potentially address these additional problems is the incorporation of a therapeutic cancer vaccine to other immunotherapeutic regimens. Studies in murine models have demonstrated that checkpoint blockade antibodies are more effective when combined with cancer vaccines than checkpoint blockade alone, even in tumors that are refractory to checkpoint blockade monotherapy [8,9]. Other studies have shown that addition of a cancer vaccine can promote epitope spreading and antigen cascade [10]; this increase in T-cell receptor (TCR) diversity has been shown to drive more potent antitumor immunity and tumor clearance [11]. Furthermore, cancer vaccines targeted to cancer-associated antigens or neoantigens have had success in the clinic and have been shown to be safe and well tolerated by patients [12,13,14].

Bintrafusp alfa is a first-in-class bifunctional fusion protein composed of the extracellular domain of the human transforming growth factor β receptor II (TGF-βRII) fused to the C-terminus of each heavy chain of an IgG1 antibody blocking PD-L1. This agent is currently being evaluated in multiple clinical studies, showing clinical activity with a confirmed objective response rate of 30.5% in patients with human papillomavirus-associated malignancies [15,16]. In a previous study, we showed that the combination of bintrafusp alfa with SX-682, a small molecule inhibitor of the chemokine receptors CXCR1 and CXCR2 that blocks signaling initiated by IL-8 and other chemokines of the CXCL family, synergizes to mediate antitumor activity in murine models of breast and lung cancer [17]. To test our hypothesis that a vaccine could help overcome some of the challenges presented by tumors that are refractory to checkpoint blockade, in the present study we investigated the effect of adding a vaccine consisting of a recombinant adenovirus serotype-5 (Ad5) vector encoding a tumor-associated antigen in combination with N-803 as an adjuvant [18] to the bintrafusp alfa/SX-682 combination. N-803 is an IL-15 super agonist that helps activate antigen-specific T cells and has shown clinical activity in combination with checkpoint blockade in non-small cell lung cancer [19,20].

Using murine models of colon and breast cancer, we demonstrate that the addition of vaccine to bintrafusp alfa/SX-682 significantly increases tumor infiltration with T cells, enhances T-cell activation and TCR diversity at the tumor site, and diversifies the number of tumor antigens being recognized by TCRs through the phenomenon of antigen cascade or epitope spreading. These results provide the rationale for the addition of cancer vaccines as integral components in combination immunotherapy approaches being evaluated in the clinic.

## 2. Materials and Methods

### 2.1. Cell Lines

BALB/c-derived 4T1 mammary carcinoma cells were obtained and cultured as recommended by the American Type Culture Collection (ATCC, Manassas, VA, USA). MC38-CEA cells were previously obtained by retroviral transduction of C57BL/6-derived MC38 colon cancer cells to overexpress human carcinoembryonic antigen (CEA) [21]. Cell lines were tested to be mycoplasma free using a MycoAlert Mycoplasma Detection Kit (Lonza, Basel, Switzerland) and used at low passage number.

### 2.2. Mice

Female BALB/c mice were obtained from the NCI Frederick Cancer Research Facility. Mice expressing human CEA on a C57BL/6 background (CEA.Tg) were generously provided by Dr. John Shively (City of Hope, Duarte, CA, USA). Mice were approximately 4 to 6 weeks old at start of experiments and were maintained under pathogen-free conditions in accordance with the Association for Assessment and Accreditation of Laboratory Animal Care guidelines. All animal studies were approved by the NIH Intramural Animal Care and Use Committee (LTIB-038) on 9 January 2018.

### 2.3. Tumor Inoculation, Treatment Schedule, and Metastasis Assay

BALB/c mice were injected in the abdominal mammary fat pad with 3 × 10^4^ 4T1 cells. CEA transgenic mice (CEA.Tg) were injected subcutaneously (s.c.) in the flank with 3 × 10^5^ MC38-CEA cells. Control diet feed or SX-682-containing feed (1428.5 mg/kg, equivalent to a dose of 200 mg/kg body weight/day; Research Diets, New Brunswick, NJ, USA) were administered to mice starting on day 7. SX-682 was provided by Syntrix Pharmaceuticals under a Cooperative Research and Development Agreement (CRADA) with the NCI. In tumor volume experiments, intraperitoneal injections (i.p.) of bintrafusp alfa (kindly provided by EMD Serono under a CRADA) were given at a dose of 200 μg per mouse starting on day 14 and every 7 days thereafter, as noted. The vaccine utilized in this study consisted of a recombinant Ad5 encoding either the tumor antigen murine Twist1, a transcription factor that is overexpressed in 4T1 tumors [22], or human CEA, which is over-expressed in MC38-CEA tumors. The Ad-vector was combined with the IL-15 super agonist N-803 as an adjuvant. The antitumor efficacy of this vaccine formulation was previously described [18], and its optimized performance was confirmed here in terms of induction of higher levels of the Th1 cytokine, TNFα, in the serum of animals in the combined Ad-vector + N-803 group versus each single agent (Figure S1). Adenovirus vaccine was administered s.c. (1 × 10^10^ viral particles) on day 7 (prime) followed by s.c. adenovirus vaccine (1 × 10^10^ viral particles) plus N-803 (1 μg, s.c.) every 7 days as noted (boosts).

Metastasis assays were performed as previously described with some modifications [17]. Lungs were harvested from 4T1 tumor-bearing mice under sterile conditions, rinsed in phosphate buffer saline (PBS), transferred to gentleMACS C tubes (Miltenyi Biotec, Waltham, MA, USA) in RPMI-1640 medium containing 5% fetal bovine serum (FBS), 5 mg/mL collagenases IV and I (Gibco, Gaithersburg, MD, USA), and 40 U/mL DNase, and dissociated using a gentleMACS tissue dissociator (Miltenyi Biotec), following the manufacturer’s recommended procedure. Cells were passed through a 70 µm filter, pelleted and washed with PBS, and resuspended in 10 mL RPMI-1640 medium supplemented with 10% FBS, 1% Na pyruvate, 1% Hepes, 1× glutamine, 1× gentamicin, and 1× penicillin-streptomycin. A 250 µL aliquot of this suspension, representing 1/40 of the total lung, was cultured in the same medium containing 60 µM 6-thioguanine for 14 days. Colonies were fixed with methanol, stained with 0.05% (*w*/*v*) methylene blue, air-dried, and counted. The number of metastases per lung was calculated as the number of colonies counted per flask ×40.

In mouse experiments quantifying TCR diversity, control or SX-682-containing feed were administered to mice starting on day 7 with i.p. injections of bintrafusp alfa given at a dose of 492 μg per mouse on days 9 and 11. The vaccine was administered s.c. (1 × 10^10^ viral particles) plus s.c. N-803 (1 μg) on day 9. Tumors were collected on day 17 post-tumor injection for subsequent TCR sequence analysis, as indicated below. Adenovirus vaccines and N-803 were kindly provided by ImmunityBio under a CRADA. In all experiments, tumors were measured every 2–3 days in two perpendicular diameters. Tumor volume = (short diameter^2^ × long diameter)/2.

### 2.4. Depletion Studies

To deplete CD8^+^ T cells from MC38-CEA tumor-bearing mice, 100 µg of anti-CD8 (clone 2.43, BioXcell, Lebanon, NH, USA) depletion antibodies were administered i.p. starting on days 5, 6, and 7 post-tumor implantation and then once per week for the duration of the experiment. Blood was obtained from all animals upon termination of the experiment to determine immune cell population depletion efficiency by flow cytometry.

### 2.5. Flow Cytometry

Prior to staining, tumors were weighed, mechanically dissociated, incubated in a shaker at 37 °C for 30 min at a speed of 300 rpm in RPMI-1640 medium containing 5% FBS, 5 mg/mL collagenases IV and I (Gibco), and 40 U/mL DNase, and then passed through a 70 µm filter as a single-cell suspension. Spleens were crushed through a 70 µm filter and red cell lysis was performed with ammonium-chloride-potassium (ACK) buffer (Gibco). All antibodies used for flow cytometry were purchased from Thermo Fisher Scientific (Waltham, MA, USA), BioLegend (San Diego, CA, USA), or BD Biosciences (San Jose, CA, USA). Cells were stained for cell surface expression in flat-bottom 96-well plates on ice in phosphate buffered saline with 2% FBS. Intracellular markers were stained using the eBioscience Foxp3/Transcription Factor Staining Buffer Set according to the manufacturer’s instructions. Fluorescently conjugated antibodies for CD45 (30-F11), CD3 (500A2), CD4 (RM4-5), CD8 (53-6.7), CD44 (IM7), CD62L (MEL14), Foxp3 (150D), Ki67 (16A8), GzmB (QA18A28), Ly6G (1A8), Ly6C (HK1.4), CD11b (M1/70), F4/80 (BM8), and CD11c (N418) were used as per the manufacturers’ instructions. LIVE/DEAD Fixable Aqua Dead Cell Stain Kit (Thermo Fisher Scientific) was used to gate on live cells. Data were acquired on an Attune NxT Flow Cytometer (Thermo Fisher Scientific) and analyzed via FlowJo (FlowJo, Ashland, OR). Immune cell subsets were defined as: CD4 = CD3^+^CD4^+^; CD8 = CD3^+^CD8^+^; T_CM_ = CD3^+^CD44^+^CD62L^+^; T_Eff&EM_ = CD3^+^CD44^+^CD62L^−^; T_regs_ = CD4^+^Foxp3^+^.

### 2.6. ELISPOT Assays

CEA.Tg mice bearing MC38-CEA tumors were fed an SX-682-containing diet starting on day 7; on days 14 and 21, mice received i.p. injections of bintrafusp alfa, with a priming vaccine dose of s.c. Ad-CEA administered on day 7 and boosting doses of Ad-CEA/N-803 vaccine on days 14 and 21. Control mice were left untreated and fed a base diet without SX-682. Splenocytes were harvested from control versus treated mice and assayed ex vivo on day 24 for antigen-dependent cytokine secretion using an IFNγ ELISPOT assay (BD Biosciences), according to the manufacturer’s instructions. Briefly, 0.5 × 10^6^ splenocytes were incubated overnight with 10 μg/mL of CEA_526–533_, p15e_604–611_, the MC38 neoepitope PTGFR, or a negative control peptide [10]. Spot-forming cells were quantified using an ImmunoSpot analyzer (Cellular Technology, Ltd, Shaker Heights, OH, USA). The amount of CD8^+^ T cells added per well was calculated by flow cytometry analysis. Data were adjusted to the number of spots/0.5 × 10^5^ CD8^+^ T cells present in the assay, subtracting the number of spots in paired wells containing the control peptide.

### 2.7. Real-Time PCR, Nanostring and TCR Analysis

Total RNA from flash-frozen tumor sections was prepared using the RNeasy Mini Kit (Qiagen, Hilden, Germany). For some experiments, RNA was then reverse-transcribed using SMARTer^®^ PCR cDNA Synthesis Kit (Takara Bio Inc, Mountain View, CA, USA) or the High-Capacity cDNA Reverse Transcription Kit (ThermoFisher Scientific) as per the manufacturer’s instructions. cDNA was amplified in triplicate using TaqMan Master Mix in an Applied Biosystems 7500 Real-Time PCR System (ThermoFisher Scientific). The following Taqman gene expression assays were used (ThermoFisher Scientific): Cd247 (Mm00446171_m1), Gzmk (Mm00492530_m1), CD8a (Mm01182107_g1), Prf1 (Mm00812512_m1), Gzmb (Mm00442837_m1), Cd3e (Mm01179194_m1), Pdcd1 (Mm00434946_m1), Tbx21 (Mm00450960_m1). NanoString analysis was performed on purified RNA samples from indicated tumors by using the PanCancer Immune Profiling Gene Expression Panel. The nSolver analysis software was used for data normalization (NanoString Technologies, Seattle, WA, USA). Further clustering and pathway analyses were performed using Ingenuity Pathway Analysis (Qiagen). To assess TCR diversity, genomic DNA was purified from whole tumor using the QIAamp DNA Mini Kit (Qiagen). TCRβ chain sequencing was then performed by Adaptive Biotechnologies and analyzed using the Immunoseq analyzer. Simpson clonality (square root of sum over all observed rearrangements of the square fractional abundances of each rearrangement) was calculated as a measurement of the observed TCRβ repertoire. The number of clones representing the top 25% of TCR sequences was used as a metric of the relative diversity of the immune response.

### 2.8. OPAL Immunofluorescence

Tumor tissue was fixed in Z-fix (Anatech, Battle Creek, MI, USA), embedded in paraffin, and sectioned onto glass slides (American HistoLabs, Gaithersburg, MD, USA). Slides were stained using the Opal 4-Color Manual IHC Kit (PerkinElmer, Waltham, MA, USA). Antigen retrieval was performed with Rodent Decloaker (BioCare Medial, Pacheco, CA, USA) antigen retrieval solution and blocked with BLOXALL Blocking Solution (Vector Laboratories, Burlingame, CA, USA). All other steps, including staining with primary and secondary antibodies and OPAL fluorophore working solution, were conducted following the manufacturer’s instructions. Antibodies used included anti-CD4 (4SM95, Invitrogen, Carlsbad, CA) and anti-CD8a (4SM16, Invitrogen). Slide scanning was performed on an Axio Scan.Z1 and Zen software (Zeiss, Oberkochen, Germany).

### 2.9. Statistical Methods

All statistical analyses were performed using GraphPad Prism V.7 for Windows (GraphPad Software, La Jolla, CA, USA). Analysis of tumor growth curves was conducted using two-way analysis of variance (ANOVA). Statistical differences between two sets of data were determined through a two-tailed Student’s *t*-test, while one-way ANOVA with Tukey’s post hoc test was used to determine statistical differences among three or more sets of data. Statistical differences between survival plots were determined using Log-rank (Mantel-Cox) test. Error bars represent SEM where noted. Asterisks indicate that the experimental *p* value is statistically significantly different from the associated controls at * *p* ≤ 0.05; ** *p* ≤ 0.01; *** *p* ≤ 0.001, **** *p* ≤ 0.0001.

## 3. Results

### 3.1. Addition of Vaccine to Checkpoint Blockade-Based Therapy Enhances Immune T-Cell Infiltration and Promotes a Th1 Tumor-Infiltrating Lymphocyte (TIL) Phenotype

**Figure 1:**
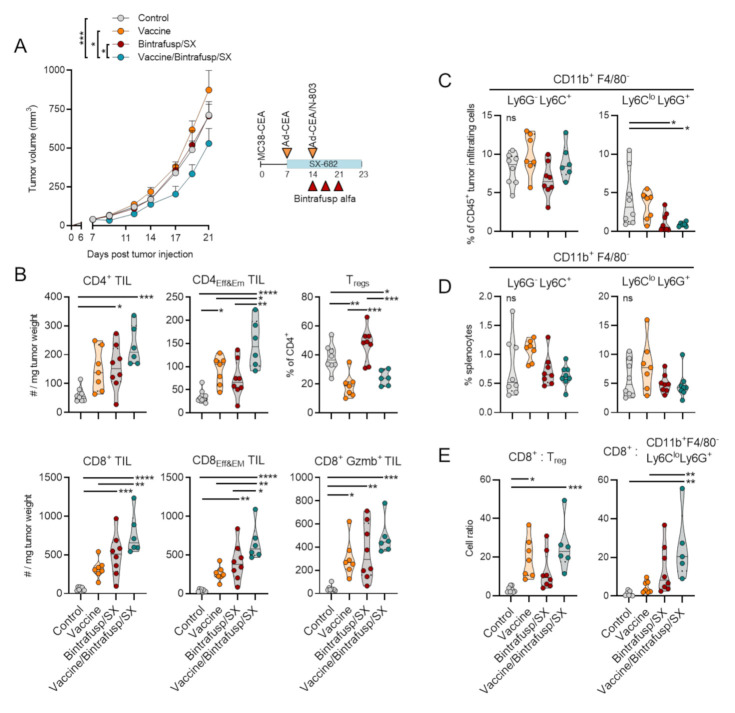
Vaccine synergizes with Bintrafusp alfa/SX-682 and increases TIL in MC38-CEA tumors. (**A**) CEA.Tg mice were injected s.c. with 3 × 10^5^ MC38-CEA in the flank. On day 7, mice were started on a control or SX-682 diet (200 mg/kg body weight/day), and on days 14, 17, and 21 mice received i.p. injections of 200 μg bintrafusp alfa. Priming vaccine dose of s.c. Ad-CEA (1 × 10^10^ viral particles) was administered on day 7 with a boosting dose of Ad-CEA/N-803 (1 × 10^10^ viral particles, N-803, 1 μg, s.c.) on day 14. Graph shows average tumor growth and error bars indicate SEM of biological replicates; *n* = 8 mice/group. * *p* ≤ 0.05; *** *p* ≤ 0.001 for two-way ANOVA in (**A**). Control indicates mice that were left untreated and fed a base diet without SX-682. Tumors (**B**,**C**) and spleens (**D**) were harvested and analyzed by flow cytometry on day 23 for lymphocytes (**B**) and myeloid cells (**C**,**D**). (**E**) Cell ratios comparing the number of cells per mg tumor weight were also calculated. Individual points represent data from one tumor. ns, not significant; * *p* ≤ 0.05; ** *p* ≤ 0.01; *** *p* ≤ 0.001; **** *p* ≤ 0.0001 for one-way ANOVA followed by Tukey’s post hoc test in (**B**–**E**). i.p. = intraperitoneal. s.c. = subcutaneous. TIL = tumor-infiltrating lymphocyte. Tregs = regulatory T cells.

The effect of adding a cancer vaccine to the combination bintrafusp alfa/SX-682 was first evaluated with CEA.Tg mice, where CEA is a self-antigen [23,24], bearing subcutaneous MC38-CEA tumors. To model a scenario where tumors do not respond to checkpoint-based immunotherapy, control feed or SX-682-containing feed were administered to mice starting on day 7, while administration of bintrafusp alfa at a low dose was delayed until day 14 to ensure response failure. In the vaccine treatment groups, mice were administered a priming vaccine dose of Ad-CEA on day 7 and a boosting dose of Ad-CEA/N-803 given on day 14 (hereafter designated “Vaccine”). As expected, the modified schedule of bintrafusp alfa plus SX-682 (Bintrafusp/SX) was unable to exert tumor control (Figure 1A). The use of vaccine as a monotherapy also failed to control tumors; the average tumor growth in the Vaccine group was statistically not different from that of the Control group (Figure 1A). Although the addition of vaccine to the Bintrafusp/SX therapy was able to induce a significant albeit modest delay in primary tumor growth in this experiment, the triple combination Vaccine/Bintrafusp/SX resulted in significant changes in the composition of the tumor immune infiltrate when compared with the other groups (Figure 1B). Overall, Vaccine/Bintrafusp/SX showed a significant enhancement of CD4^+^ and CD8^+^ T cells characterized by an effector and effector-memory phenotype (CD4_Eff&Em_ and CD8_Eff&Em_ TIL) above the levels achieved in the Vaccine monotherapy, Bintrafusp/SX, and Control groups (Figure 1B). Also remarkable was the ability of vaccine to decrease the percentage of regulatory T cells (T_regs_) in the CD4^+^ TIL population, compared to the Control and Bintrafusp/SX groups (Figure 1B). Previously, we demonstrated that Bintrafusp/SX therapy can significantly reduce tumor infiltration with suppressive granulocytic myeloid-derived suppressor cells (G-MDSC), defined as CD11b^+^F4/80^−^Ly6C^lo^Ly6G^+^, an effect attributed to the ability of SX-682 to block the CXCR1/2-mediated migration of G-MDSC into the tumor. The effect was not observed with monocytic MDSC, defined as CD11b^+^F4/80^−^Ly6G^−^Ly6C^+^. Here, CD11b^+^F4/80^−^Ly6C^lo^Ly6G^+^ cells were significantly reduced in the tumors of mice treated with both Bintrafusp/SX and Vaccine/Bintrafusp/SX, an effect that was not observed with CD11b^+^F4/80^−^Ly6G^−^Ly6C^+^ fractions (Figure 1C). Neither fraction of myeloid cells was altered in the spleen of mice in any of the treatment groups (Figure 1D). As shown in Figure 1E, only Vaccine/Bintrafusp/SX treatment induced a significant increase in the ratio of CD8^+^ TIL to both T_regs_ and CD11b^+^F4/80^−^Ly6C^lo^Ly6G^+^ cells in the TME compared to Control mice.

**Figure 2:**
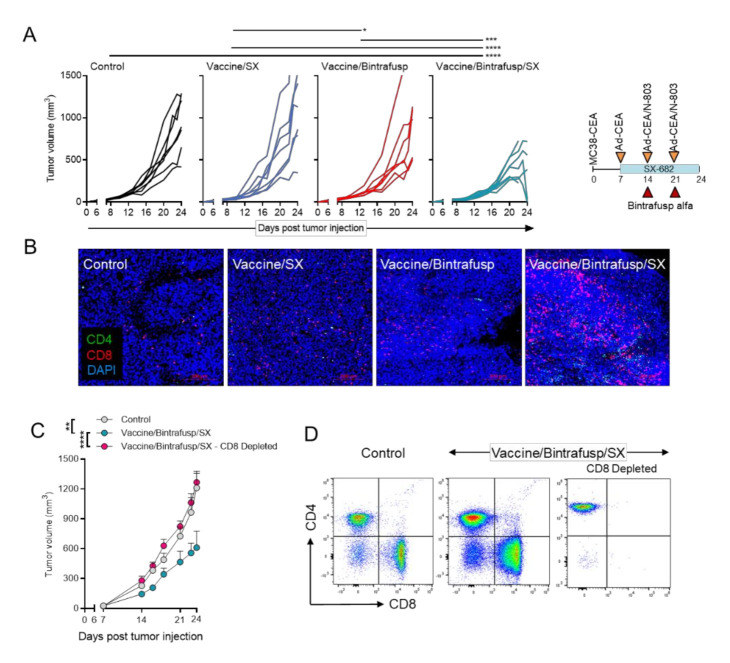
Vaccine combination immunotherapy is dependent on CD8^+^ TIL. (**A**) CEA.Tg mice were injected s.c. with 3 × 10^5^ MC38-CEA in the flank. On day 7, mice were started on a control or SX-682 diet (200 mg/kg body weight/day). On days 14 and 21, mice received i.p. injections of 200 μg bintrafusp alfa. A priming vaccine dose of s.c. Ad-CEA (1 × 10^10^ viral particles) was administered on day 7 with a boosting dose of Ad-CEA/N-803 vaccine on days 14 and 21 (1 × 10^10^ viral particles, N-803, 1 μg, s.c.). Shown are the individual tumor growths for mice in the Control, Vaccine/SX, Vaccine/Bintrafusp, and Vaccine/Bintrafusp/SX groups; *n* = 7 mice/group. Control indicates mice that were left untreated and fed a base diet without SX-682. (**B**) Representative images of indicated tumors stained for CD4^+^ (green) and CD8^+^ (red) T cells and DAPI (blue) by immunofluorescence. (**C**) MC38-CEA tumor-bearing CEA.Tg mice received Vaccine/Bintrafusp/SX as in (**A**). Additionally, mice receiving Vaccine/Bintrafusp/SX also received depleting antibodies for CD8^+^ cells starting on day 5; *n* = 7 (Control and Vaccine/Bintrafusp/SX – CD8 Depleted) or 5 (Vaccine/Bintrafusp/SX) mice/group. (**D**) Flow profiles confirming efficacy of CD8 depletion antibodies from (**C**). Error bars indicate SEM of biological replicates. * *p* ≤ 0.05; ** *p* ≤ 0.01; *** *p* ≤ 0.001; **** *p* ≤ 0.0001 for two-way ANOVA in (**A**,**C**). i.p. = intraperitoneal. s.c. = subcutaneous. TIL = tumor-infiltrating lymphocyte.

To understand whether both bintrafusp alfa and SX-682 were needed for the anti-tumor efficacy of the combination Vaccine/Bintrafusp/SX, in the next study we also evaluated the addition of vaccine to SX-682 (Vaccine/SX) or bintrafusp alfa alone (Vaccine/Bintrafusp). In this experiment, an additional boosting dose of vaccine was administered on day 21. While the growth of MC38-CEA tumors was not delayed with Vaccine/SX or Vaccine/Bintrafusp combinations, there was a significant delay in tumor growth in the Vaccine/Bintrafusp/SX group (Figure 2A). Interestingly, some tumors began to completely regress in the Vaccine/Bintrafusp/SX group immediately after the final dose of vaccine plus bintrafusp alfa administered on day 21. Sections of tumor tissue stained by immunofluorescence revealed high levels of infiltrating CD4^+^ and CD8^+^ T cells in the Vaccine/Bintrafusp/SX group that were distributed uniformly throughout the tumors, compared to the other groups (Figure 2B).

The importance of the CD8^+^ T-cell fraction for the effectiveness of the multimodal therapy was evaluated with CEA.Tg mice bearing MC38-CEA tumors that were either left untreated and fed a base diet without SX-682 (Control group), treated with Vaccine/Bintrafusp/SX multimodal therapy, or treated with multimodal therapy with simultaneous depletion of CD8^+^ T cells (Vaccine/Bintrafusp/SX – CD8 Depleted group, Figure 2C,D). As shown in Figure 2C, depletion of CD8^+^ T cells completely abrogated the antitumor efficacy of Vaccine/Bintrafusp/SX treatment. The triple combination also had a modest yet significant effect on the survival of MC38-CEA tumor-bearing mice over that of Bintrafusp/SX-treated or Control mice (Figure S2).

**Figure 3:**
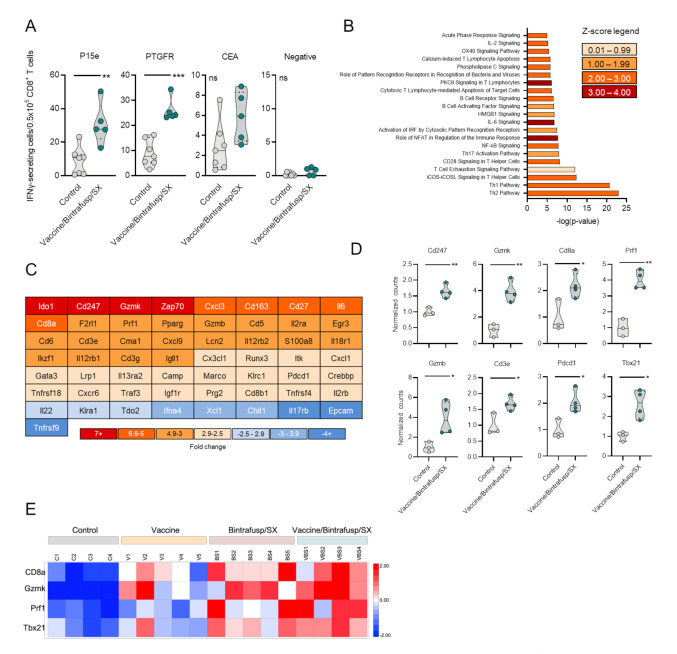
Immune activation signature observed in MC38-CEA tumors treated with Vaccine/Bintrafusp/SX combination. CEA.Tg mice were injected s.c. with 3 × 10^5^ MC38-CEA in the flank. On day 7, mice were started on a control or SX-682 diet (200 mg/kg body weight/day). On days 14 and 21, mice received i.p. injections of 200 μg bintrafusp alfa. A priming vaccine dose of s.c. Ad-CEA was administered on day 7 (1 × 10^10^ viral particles) with a boosting dose of Ad-CEA/N-803 vaccine on days 14 and 21 (1 × 10^10^ viral particles, N-803, 1 μg, s.c.). (**A**) IFNγ ELISPOT analysis of spleens collected on day 24 from Control and Vaccine/Bintrafusp/SX-treated mice against MC38-CEA tumor antigens. Control indicates mice that were left untreated and fed a base diet without SX-682; n = 7 (Control) or 5 (Vaccine/Bintrafusp/SX) mice/group. Tumors collected on day 24 were used for RNA preparation and NanoString analysis as described in the Materials and Methods. Shown in (**B**) is an Ingenuity Pathway Analysis performed on genes that were found to be up- or down-regulated more than 2-fold in Vaccine/Bintrafusp/SX-treated tumors compared to Control tumors; *n* = 3 mice/group. (**C**) Heat map of genes differentially expressed >2.5-fold in Vaccine/Bintrafusp/SX-treated tumors compared to Control tumors; *n* = 3 mice/group. (**D**) Real-time PCR analysis confirming selected genes upregulated in Vaccine/Bintrafusp/SX-treated tumors compared to Control tumors; *n* = 3 (Control) or 4 (Vaccine/Bintrafusp/SX) mice/group. Individual points represent data from one tumor. ns, not significant; * *p* ≤ 0.05; ** *p* ≤ 0.01; *** *p* ≤ 0.001 for two-tailed Student’s *t*-test in (**A**,**D**). (**E**) Heat map expression of indicated genes in MC38-CEA tumors treated as per the schedule of administration in Figure 1. Tumor RNA was prepared at day 23; RNA expression of indicated genes was evaluated by real-time PCR as described in the Materials and Methods.

It has been previously reported that combination therapy consisting of vaccine and various immune modulatory agents, including immune checkpoint blockade, can enhance antitumor immunity by diversifying the number of tumor antigens being recognized by TCRs through the phenomenon of antigen cascade or epitope spreading [10]. In this study, splenocytes from Control and Vaccine/Bintrafusp/SX-treated mice were evaluated for potential epitope spreading by quantifying on an ELISPOT assay the number of CD8^+^ T cells specific for CEA, the MC38-neoantigen PTGFR [10], or P15e, compared to a negative control peptide (Figure 3A). While there was a modest enhancement of the number of T cells specific for CEA in the spleens of vaccinated mice (~2-fold increase), high numbers of both PTGFR-specific and P15e-specific T cells were observed in the Vaccine/Bintrafusp/SX-treated mice, compared to the Control group (2.9-fold and 3.6-fold, respectively) (Figure 3A).

To understand how the combination of these agents restructures the immune profile of the TME in Vaccine/Bintrafusp/SX-treated tumors, NanoString gene expression analysis was performed on whole tumor tissue-derived RNA. Table 1 lists genes that were found to be up- or down-regulated more than 2.0-fold in Vaccine/Bintrafusp/SX-treated mice compared to Control tumors. Ingenuity Pathway Analysis demonstrated an upregulation of many immune-specific canonical pathways, with Th1 and Th2 being the two most significantly upregulated pathways (Figure 3B) in Vaccine/Bintrafusp/SX versus Control tumors. In addition, strong upregulation of inducible T-cell costimulator (ICOS) signaling, nuclear factor of activated T cells (NFAT) regulation, CTL-mediated apoptosis of target cells, and CD28 signaling were observed in tumors treated with the multimodal therapy Vaccine/Bintrafusp/SX versus Control. Figure 3C shows genes that were up- or down-regulated >2.5-fold in the triple combination group, with some of them being confirmed by PCR analysis in tumors of mice treated with Vaccine/Bintrafusp/SX versus Control (Figure 3D). There was a significant upregulation of Cd3e, Cd8a, Tbx21, Pdcd1, Cd247, and genes encoding for the effector molecules, Prf1, Gzmb, and Gzmk, suggesting a highly cytotoxic phenotype in TIL isolated from Vaccine/Bintrafusp/SX-treated tumors. Additional PCR analysis of expression of CD8a, Tbx21, Gzmk, and Prf1 mRNA was conducted in individual tumors from the Control, Vaccine, Bintrafusp/SX and Vaccine/Bintrafusp/SX groups. While vaccine used as monotherapy induced only a modest upregulation of these genes in some of the tumors compared with Control tumors, a stronger upregulation was observed in the Bintrafusp/SX group, though the level of upregulation was variable among genes and across tumor samples (Figure 3E). Supporting the benefit of adding all agents together, tumors in the Vaccine/Bintrafusp/SX group exhibited a more robust upregulation of all four genes in the majority of samples evaluated (Figure 3E). These data indicated that addition of vaccine can further enhance immune infiltration and activation above the induction mediated by blockade of PD-L1, TGF- β and CXCR1/2.

### 3.2. Addition of Vaccine to Checkpoint Blockade-Based Therapy Enhances Immune T-Cell Activation and TCR Diversity

**Figure 4:**
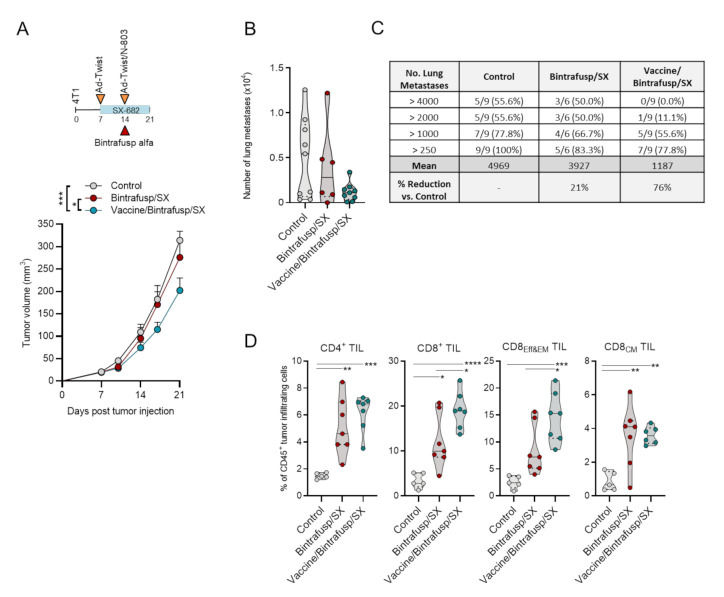
Vaccine synergizes with Bintrafusp alfa and SX-682 and increases TIL in 4T1 tumors. (**A**) BALB/c mice bearing 4T1 tumors in the mammary fat pad received control or SX-682 diet on day 7 (200 mg/kg body weight/day), with a priming vaccine dose of s.c. Ad-Twist (1 × 10^10^ viral particles). On day 14, mice received an i.p. injection of 200 μg bintrafusp alfa with a boosting vaccine dose of Ad-Twist/N-803 (1 × 10^10^ viral particles, N-803, 1 μg, s.c.). Graph shows average tumor growth and error bars indicate SEM of biological replicates; *n* = 6 (Control) or 7 (Bintrafusp/SX, Vaccine/Bintrafusp/SX) mice/group. Control indicates mice that were left untreated and fed a base diet without SX-682. * *p* ≤ 0.05; *** *p* ≤ 0.001 for two-way ANOVA. (**B**) Number of metastases quantified in the lungs of 4T1 tumor-bearing mice on day 21; individual points represent data from one mouse. (**C**) Table depicting the number and percentage of mice with the indicated range of lung metastases in each group, the mean number of metastases in each group, and the % reduction of the mean in each group vs. the Control group. Data are pooled from 2 independent experiments. (**D**) Tumors were harvested and analyzed by flow cytometry on day 21. Individual points represent data from one tumor. * *p* ≤ 0.05; ** *p* ≤ 0.01; *** *p* ≤ 0.001; **** *p* ≤ 0.0001 for one-way ANOVA followed by Tukey’s post hoc test. i.p. = intraperitoneal. s.c. = subcutaneous. TIL = tumor-infiltrating lymphocyte.

To corroborate the results in a different tumor model, a single dose of bintrafusp alfa in combination with SX-682 was given to 4T1 tumor-bearing mice which, as expected, failed to control tumor growth (Bintrafusp/SX, Figure 4A). In this mammary carcinoma model, vaccine was administered as a priming dose of Ad-Twist on day 7 with a boosting vaccine on day 14 consisting of Ad-Twist plus N-803. Twist1, a transcription factor that drives metastasis, was identified and characterized as a targetable “self” tumor-associated antigen in 4T1 tumor cells [22]. Addition of vaccine to Bintrafusp/SX therapy induced only a modest delay in primary tumor growth (Vaccine/Bintrafusp/SX, Figure 4A), and a trend towards reduced number of lung metastases (Figure 4B), with a 76% reduction of metastases in the Vaccine/Bintrafusp/SX group compared with the Control (Figure 4C). Two caveats with these results, however, are the low number of mice evaluated in each group, and the reduction of primary tumor volume in the Vaccine/Bintrafusp/SX group that could directly impact the number of disseminated cells.

Similar to the results observed with MC38-CEA tumors, addition of vaccine had a marked impact on the composition of 4T1 primary tumor T-cell infiltrates. As shown in Figure 4D, flow cytometry analysis of tumors collected at 1 week post-bintrafusp alfa ± vaccine administration (day 21 post-tumor injection) revealed significantly higher frequencies of CD8^+^ T cells characterized by an effector and effector-memory phenotype (CD8_Eff&EM_) in the Vaccine/Bintrafusp/SX group compared with the Bintrafusp/SX group or Control tumors. In contrast, the frequency of CD4^+^ T cells and central memory CD8^+^ T cells (CD8_CM_) were similar among the two treatment groups, irrelevant of vaccine. In agreement with the flow cytometry data, immunofluorescence-based analysis of TIL in sections of Formalin-Fixed Paraffin-Embedded (FFPE) tumor tissues (Figure S3A) showed large clusters of CD4^+^ and CD8^+^ T cells homogenously distributed throughout the tumor in Vaccine/Bintrafusp/SX-treated tumors and not solely contained to the tumor boundaries. Consistent with previous findings, immune subset profiling of Vaccine/Bintrafusp/SX-treated tumors also revealed a significant decrease in the frequency of tumor-infiltrating CD11b^+^F4/80^-^Ly6G^+^Ly6C^lo^ myeloid cells and CD11b^+^F4/80^hi^ macrophages, together with a marked increase of CD4^+^ and CD8^+^ T cells (Figure S3B). Additionally, no adverse events or toxicity were observed with the total combination of therapeutics. These results suggested that addition of a prime-boost vaccine to a checkpoint blockade-based immunotherapy can further enhance frequency of effector T lymphocytes in the TME.

**Figure 5:**
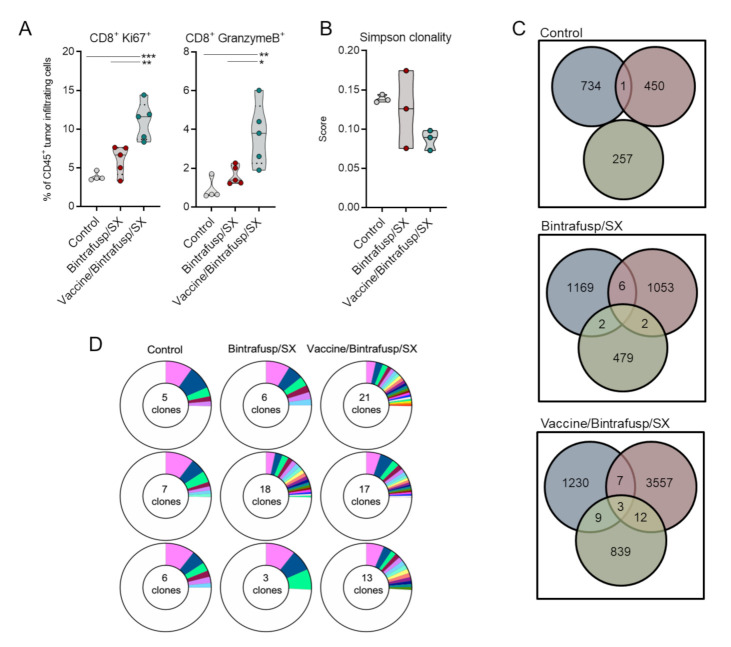
Vaccine enhances activation and TCR diversity of TIL when incorporated into combination immunotherapy in the 4T1 carcinoma model. (**A**) BALB/c mice bearing 4T1 tumors in the mammary fat pad received control or SX-682 diet on day 7 (200 mg/kg body weight/day), with a priming vaccine dose of s.c. Ad-Twist (1 × 10^10^ viral particles). On day 14, mice received an i.p. injection of 200 μg bintrafusp alfa with a boosting vaccine dose of Ad-Twist/N-803 (1 × 10^10^ viral particles, N-803, 1 μg, s.c.). Graphs show immune subsets determined by flow cytometry analysis of tumors at day 21. Individual points represent data from one tumor. * *p* ≤ 0.05; ** *p* ≤ 0.01; *** *p* ≤ 0.001 for one-way ANOVA followed by Tukey’s post hoc test. (**B**) Simpson clonality score for individual tumor samples in each indicated group determined as indicated in the Materials and Methods. (**C**) Number of productive TCRβ rearrangements per individual tumor in the indicated groups, showing the number of overlapping TCRβ sequences among individuals. (**D**) The number of TCRβ clones comprising the top 25% of detected sequences. *n* = 3 mice/group. i.p. = intraperitoneal. s.c. = subcutaneous. TCR = T-cell receptor. TIL = tumor-infiltrating lymphocyte.

The quality of the T-cell infiltrates in 4T1 tumors of Bintrafusp/SX ± vaccine-treated mice was further evaluated. Intracellular flow cytometry-based analysis of tumor-infiltrating T cells from Vaccine/Bintrafusp/SX-treated mice revealed significantly higher frequencies of proliferative (CD8^+^ Ki67^+^) and cytotoxic (CD8^+^ Granzyme B^+^) TIL compared to tumors in the Bintrafusp/SX and Control groups (Figure 5A). TCRβ sequencing analysis was also performed on whole tumor lysates from 3 individual tumors per group; addition of vaccine to Bintrafusp/SX resulted in reduced clonality (Figure 5B) and expanded the T-cell repertoire compared with Control and Bintrafusp/SX-treated tumors, with an average of 481 ± 240, 907 ± 372, and 1897 ± 1469 productive TCRβ rearrangements in the Control, Bintrafusp/SX and Vaccine/Bintrafusp/SX groups, respectively (Figure 5C).

**Figure 6:**
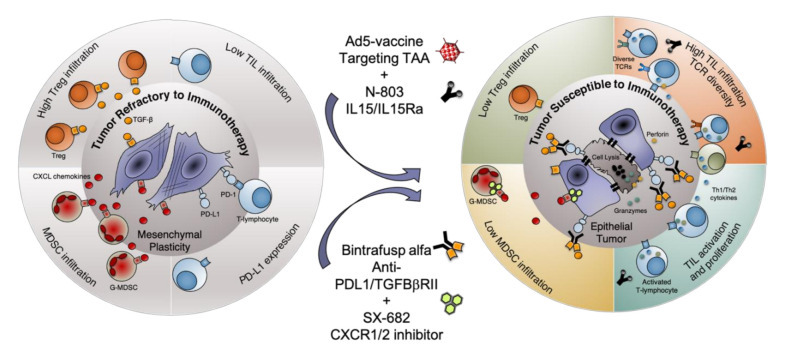
Schematic representation of the mechanism of action of the combination Ad5-vaccine, N-803, Bintrafusp alfa and SX-682. G-MDSC = granulocytic myeloid-derived suppressor cells. TCR = T-cell receptor. TIL = tumor-infiltrating lymphocyte. Tregs = regulatory T cells.

In addition, analysis of sequence similarities revealed a higher number of TCRβ sequences shared among tumors in the Vaccine/Bintrafusp/SX > Bintrafusp/SX > Control group, as shown by the numbers in the regions of intersection. Analysis of the top 25% of TCRβ sequences present in tumors from 3 mice in each group revealed a more diversified TCR repertoire in the Vaccine/Bintrafusp/SX-treated mice (Figure 5D) comprising 21, 17, and 13 clones per individual, while tumors from Control and Bintrafusp/SX-treated mice contained 5, 7, 6 and 6, 18, and 3 different TCRβ clones, respectively. These data indicated that the addition of a vaccine consisting of Ad-vector plus N-803 adjuvant to bintrafusp alfa plus SX-682 therapy has the potential to increase the proliferation and cytotoxic functionality of tumor-infiltrating CD8^+^ T cells, while promoting a more diversified TCR repertoire in the tumor (Figure 6).

## 4. Discussion

In this study, we demonstrate the effect of adding a cancer vaccine to immune checkpoint blockade therapy. Our data show that a vaccine consisting of a recombinant adenovirus with a target antigen transgene coupled with an IL-15 super agonist adjuvant is able to contribute to checkpoint-based immunotherapy by increasing T-cell migration to the tumor, enhancing T-cell activation and cytotoxicity, and promoting TCR diversity and antigen cascade.

The mechanism of action and immunological benefits of both bintrafusp alfa and SX-682 have been extensively studied as monotherapies and in combination by our group and others. Bintrafusp alfa, designed as a checkpoint inhibitor and to “trap TGF-β” in the TME, has been shown to promote T- and NK-cell killing of tumor cells, promote antibody-dependent cell cytotoxicity, revert TGF-β-induced epithelial-mesenchymal phenotypic changes in cancer cells (tumor cell plasticity), and delay tumor growth in numerous mouse models of cancer [15,25,26,27]. There are numerous ongoing clinical studies of bintrafusp alfa in patients with a variety of cancer types, with several of these studies investigating its use in combination with other immunotherapies, chemotherapy or radiation [15]. SX-682 is a small molecule inhibitor that allosterically binds to the CXCR1 and CXCR2 receptors to irreversibly inhibit downstream signaling from CXC family ligands CXCL1-3 and CXCL5-8. One of the most notable CXCR1/2 ligands, IL-8 (CXCL8), is a known inducer of tumor cell plasticity, attractant of suppressive myeloid-derived suppressor cells to the tumor, and correlates with failure of treatment in numerous cancer types, including failure to checkpoint inhibitor therapy [28,29,30,31]. SX-682 has been shown to inhibit tumor growth, block migration of G-MDSC to tumors in vivo, and decrease markers of tumor cell plasticity in human xenografts and murine tumors [17,32,33], and is currently undergoing clinical evaluation in several clinical trials [29]. In a previous study, we demonstrated that the combination of bintrafusp alfa and SX-682 reduces mesenchymal tumor features and increases epithelial protein expression in murine models of breast and lung cancer, reduces tumor infiltration with G-MDSC, and enhances T-cell infiltration and activation in tumors [17].

Tumor immunologists have been attempting to develop highly specific yet off-the-shelf immune activating vaccines for the treatment of cancer patients prior to the immune checkpoint blockade revolution. These vaccines often targeted tumor-associated antigens and were combined with immune-activating adjuvants or costimulatory molecules to promote T-cell infiltration into tumors and kick-start antitumor immunity [8,34,35]. More recent studies have also found efficacy with the use of neoantigen-based vaccines and irradiated cancer cell vaccines. However, the subsequently activated T-cell population can still be rapidly inhibited by immune checkpoint pathways or immune suppressive cells once arriving to the tumor. Additionally, many tumor types with low degree of T-cell infiltration which respond poorly to immunotherapy such as pancreatic, colon, and prostate cancers upregulate additional immune suppressive mechanisms including TGF-β, MDSC, and mesenchymal features [36,37,38,39]. In this study, we lowered the dose and delayed the administration of bintrafusp alfa in combination with SX-682 with the idea of preventing antitumor activity to mimic the situation of non-responsive tumors. We were able to demonstrate that the addition of vaccine in this context promoted further T-cell infiltration and activation, and enhanced TCR diversity in the tumor above what was induced by bintrafusp alfa/SX-682 treatment (Figure 6). We also showed here that addition of vaccine further enhanced the expression of genes indicative of immune activation and T-cell infiltration in the TME (CD8a, Tbx21, Gmzk, Prf1). These data are in agreement with the flow cytometric analysis of MC38-CEA tumors, which demonstrated an increased number of infiltrating CD4^+^ effector/effector memory T cells as well as CD8^+^ effector/effector-memory T cells in Vaccine/Bintrafusp/SX-treated tumors versus tumor in the Bintrafusp/SX group. Similarly, infiltration with CD8^+^ effector/effector-memory T cells was significantly enhanced in 4T1 tumors treated with Vaccine/Bintrafusp/SX versus Bintrafusp/SX treatment. Additionally, increased proliferation and cytolytic effect of T cells was observed in the TME of Vaccine/Bintrafusp/SX-treated 4T1 tumors, denoted by a higher percentage of CD8^+^ T cells positive for Ki67 or Granzyme B, compared with tumors in the Bintrafusp/SX group.

Analysis of splenocytes via ELISPOT assay also revealed epitope spreading in the Vaccine/Bintrafusp/SX-treated mice, with an increase in the number of T cells specific for antigens found in the tumor but not in the vaccine (PTGFR and P15e), compared with the Control group. One could hypothesize that these activated, tumor-specific T cells from spleens of Vaccine/Bintrafusp/SX-treated mice could mediate some degree of tumor control if adoptively transferred into MC38-CEA tumor-bearing mice; however, such experiments would not be able to reveal the full potential of this combination immunotherapy, which relies on tumor-localized effects mediated by SX-682 and bintrafusp alfa. As we have previously shown, inhibition of CXCR1/2 via SX-682 significantly reduces the migration of suppressive CXCR2^+^ G-MDSC into tumors. At the same time, SX-682 directly affects the phenotype of the tumor cells resulting in reduced mesenchymal features which, in turn, improves tumor susceptibility to immune-mediated lysis [17]. Similarly, bintrafusp alfa is able to mediate neutralization of PD-L1 and TGF-β in the TME, leading to alleviation of local tumor immunosuppression mediated by both pathways, including the reversion of tumor mesenchymal features for improved susceptibility to immune attack [15,17].

Despite increased infiltration of tumors with activated T cells and increased numbers of tumor-specific T cells in the Vaccine/Bintrafusp/SX group, the treatment schedules investigated here did not result in a significant number of tumor cures. We hypothesize that this could have been due to various factors, including the limited therapeutic window in which the human drugs employed here could be administered to immune competent mice without production of anti-drug antibodies. Another possibility is the very rapid tumor growth characteristic of the two murine models utilized in this study, combined with a delayed initiation of therapy, which limited time for treatment. Notably, in the clinical setting, multiple agents can be administered continuously with optimal dosing over an extended period of time for maximum benefit, as in the case of the combination of Adenoviral-based vaccines, N-803, and bintrafusp alfa currently being tested in the clinic [40]. Alternatively, other mechanisms of immune suppression may have limited tumor control in the combination group, even in the presence of activated, infiltrating T cells. Interestingly, one of the genes most upregulated in MC38-CEA tumors treated with Vaccine/Bintrafusp/SX was Ido1, suggestive of the possibility of adding an IDO inhibitor to this therapeutic regimen. Overall, the combination Vaccine/Bintrafusp/SX therapy was more effective at controlling MC38 compared with 4T1 tumor growth, an effect that could be related to the higher mutational burden and neoepitope expression in MC38 versus 4T1 tumors.

In conclusion, this study highlights the mechanistic synergy between vaccine and combination checkpoint immunotherapy and provides rationale for an ongoing clinical trial combining a cancer vaccine with bintrafusp alfa plus SX-682 therapy in patients with advanced solid tumors (NCT04574583).

## Figures and Tables

**Table 1:** Genes that were found to be up- or down-regulated more than 2.0-fold in Vaccine/Bintrafusp/SX-treated mice compared to Control tumors.

Gene	Fold Change	Gene	Fold Change	Gene	Fold Change
*Ido1*	10.13	*Tnfrsf18*	2.59	*Csf1*	2.13
*Cd247*	8.73	*Cxcr6*	2.53	*Pou2f2*	2.13
*Gzmk*	8.56	*Traf3*	2.53	*Igf2r*	2.12
*Zap70*	7.41	*Igf1r*	2.52	*Itgal*	2.11
*Cxcl3*	6.93	*Prg2*	2.52	*Notch1*	2.11
*Cd163*	6.77	*Cd8b1*	2.51	*Pnma1*	2.11
*Cd27*	6.75	*Tnfrsf4*	2.51	*Hc*	2.1
*Il6*	6.75	*Il2rb*	2.5	*Cmah*	2.09
*Cd8a*	6.7	*Nfatc2*	2.49	*Inpp5d*	2.09
*F2rl1*	5.76	*Dmbt1*	2.47	*Cxcl2*	2.08
*Prf1*	5.37	*CD209e*	2.46	*Smad3*	2.07
*Pparg*	5.35	*Cxcl5*	2.46	*Angpt1*	2.06
*Gzmb*	5.21	*Ccl3*	2.45	*Tfe3*	2.05
*Cd5*	5.16	*Itga4*	2.43	*Fcer1a*	2.04
*Il2ra*	4.85	*Polr2a*	2.43	*Masp1*	2.04
*Egr3*	4.33	*Egr1*	2.42	*Bst1*	2.02
*Cd6*	4.01	*Gbp5*	2.42	*Erbb2*	2.02
*Cd3e*	3.8	*Sap130*	2.39	*Rel*	2.02
*Cma1*	3.73	*Tlr9*	2.36	*Tapbp*	2.02
*Cxcl9*	3.59	*Nlrc5*	2.35	*Tirap*	2.01
*Lcn2*	3.56	*Il25*	2.33	*Sdha*	2.01
*Il12rb2*	3.42	*Pin1*	2.33	*Cr2*	2
*S100a8*	3.42	*C8b*	2.3	*Cd7*	−2.01
*Il18r1*	3.34	*Icos*	2.28	*Il17b*	−2.03
*Ikzf1*	3.18	*Lyve1*	2.28	*Aire*	−2.08
*Il12rb1*	3.18	*Elk1*	2.27	*Tnfrsf17*	−2.15
*Cd3g*	3.17	*Ep300*	2.27	*Ms4a1*	−2.21
*Igll1*	3.16	*Gbp2b*	2.23	*Cfd*	−2.43
*Cx3cl1*	2.86	*C4b*	2.22	*Il12a*	−2.48
*Runx3*	2.84	*Crp*	2.22	*Il22*	−2.5
*Itk*	2.83	*Nfatc3*	2.22	*Klra1*	−2.54
*Cxcl1*	2.82	*Cxcl13*	2.21	*Tdo2*	−2.82
*Gata3*	2.8	*Atm*	2.2	*Ifna4*	−3.04
*Lrp1*	2.79	*Il6ra*	2.2	*Xcl1*	−3.17
*Il13ra2*	2.76	*Tnfrsf11b*	2.2	*Chit1*	−3.7
*Camp*	2.67	*Fasl*	2.19	*Il17rb*	−3.94
*Marco*	2.67	*Jun*	2.19	*Epcam*	−4.85
*Klrc1*	2.65	*Ddx58*	2.18	*Tnfrsf9*	−5.48
*Pdcd1*	2.63	*Il18rap*	2.15		
*Crebbp*	2.62	*Tigit*	2.14		

## Data Availability

The data presented in this study will be provided upon reasonable request.

## Supplementary Materials

The following are available online at https://www.mdpi.com/2072-6694/13/5/968/s1, Figure S1: Optimization of the combination Ad-CEA plus N-803, Figure S2: Survival of CEA.Tg mice bearing MC38-CEA tumors in response to indicated treatments, Figure S3: Multimodal therapy effect on 4T1 tumor immune cell infiltration.
