# Supplementary material for: Vaccine Increases the Diversity and Activation of Intratumoral T Cells in the Context of Combination Immunotherapy
Source: Cancers (Basel). 2021 Feb 25;13(5):968. doi: 10.3390/cancers13050968 (PMC7956439; doi:10.3390/cancers13050968)
Supplement: Supplementary file 1 [file cancers-13-00968-s001.pdf]

cancers

Supplementary Materials

Vaccine Increases the Diversity and Activation of Intratumoral T Cells in the Context of Combination Immunotherapy

Lucas A. Horn, Kristen Fousek, Duane H. Hamilton, James W. Hodge, John A. Zebala, Dean Y. Maeda, Jeffrey Schlom and Claudia Palena

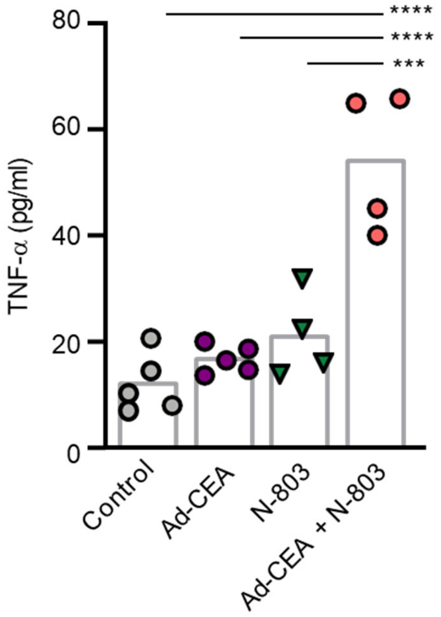

Figure S1. Optimization of the combination Ad-CEA plus N-803. Female C57BL/6-CEA.Tg mice (10- to 16-week-old) were injected with MC38-CEA tumors. Mice were administered either Ad-CEA alone on days 7, 14, and 21 post-tumor implantation, N-803 alone on days 10 and 17, or the combination of the two agents. On day 25 post-tumor implantation, sera were collected ($n = 4$–5/group) and TNFα levels quantified. Individual points represent data from one mouse. * $p \leq 0.05$; ** $p \leq 0.01$; *** $p \leq 0.001$; **** $p \leq 0.0001$ for one-way ANOVA followed by Tukey's post hoc test.

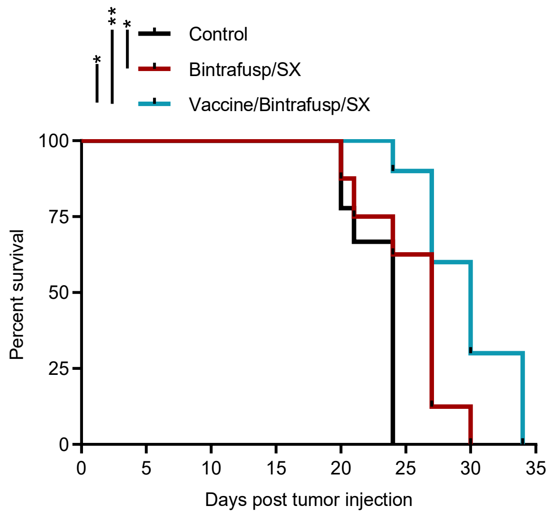

Figure S2. CEA.Tg mice were injected s.c. with 3 × 10^5 MC38-CEA in the flank. On day 7, mice were started on a control or SX-682 diet, and on days 14 and 21 mice received i.p. injections of 200 µg bintrafusp alfa. Priming vaccine dose of s.c. Ad-CEA was administered on day 7 with boosting doses of Ad-CEA/N-803 on days 14 and 21. Survival curves show percent survival; $n = 7$ mice/group. * $p \leq 0.05$; ** $p \leq 0.01$ for Log-rank (Mantel-Cox) test.

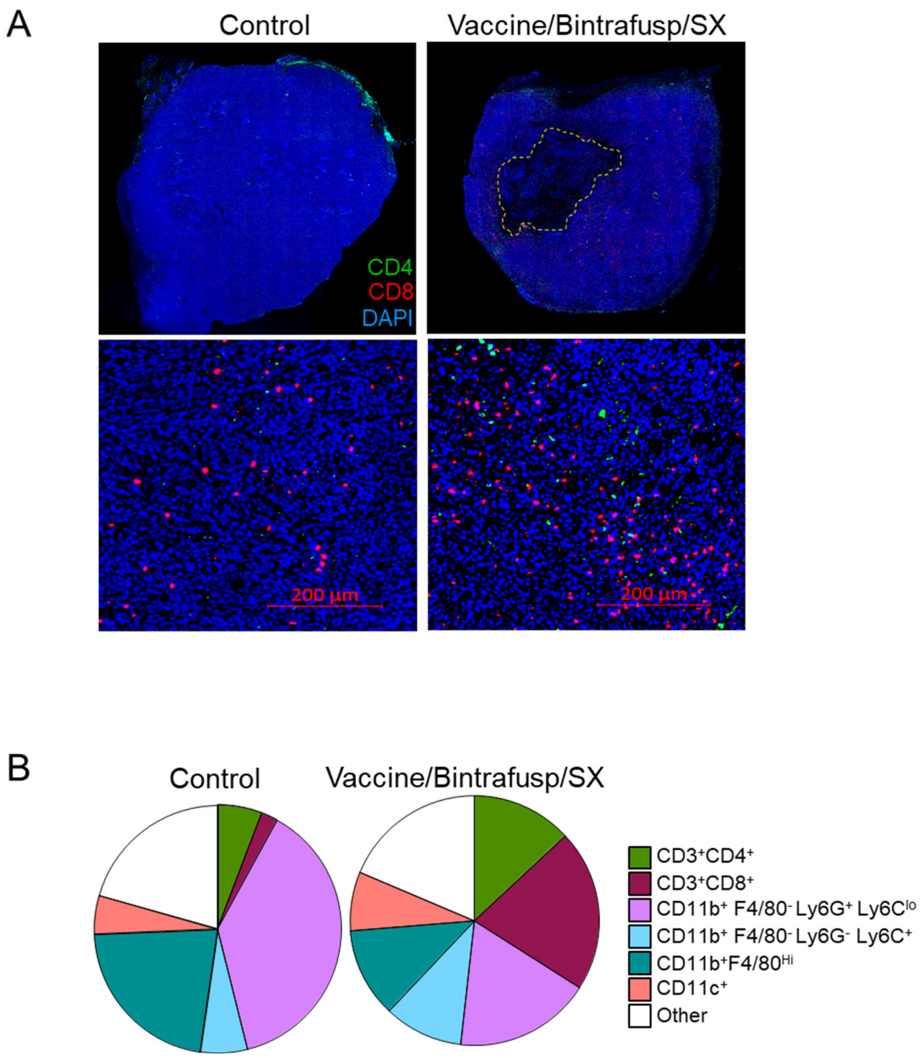

Figure S3. (**A**) Representative images of tumors stained for CD4+ (green) and CD8+ (red) T cells and DAPI (blue) by immunofluorescence. Upper panels show images for the entire tumor, with area of central necrosis delineated by a dashed line; lower panels showed a magnified area. (**B**) Flow cytometry analysis of Control and Vaccine/Bintrafusp/SX-treated tumors comparing average frequencies of tumor infiltrating immune subsets per total CD45+; $n = 5$ (Control), $n = 6$ (Vaccine/Bintrafusp/SX).
